# Similar glycaemic control and risk of hypoglycaemia with patient- versus physician-managed titration of insulin glargine 300 U/mL across subgroups of patients with T2DM: a post hoc analysis of ITAS

**DOI:** 10.1007/s00592-021-01675-0

**Published:** 2021-02-14

**Authors:** Andrea Giaccari, R. C. Bonadonna, R. Buzzetti, G. Perseghin, D. Cucinotta, C. Fanelli, A. Avogaro, G. Aimaretti, M. Larosa, V. Pagano, G. B. Bolli

**Affiliations:** 1 Fondazione Policlinico Universitario A. Gemelli IRCCS, Rome and Università Cattolica del Sacro Cuore, Rome, Italy; 2 Division of Endocrinology and Metabolic Diseases and Department of Medicine and Surgery, University of Parma and AOU of Parma, Parma, Italy; 3 Sapienza University of Rome, Piazzale Aldo Moro, 5, 00185 Rome, RM Italy; 4 University of Milan Bicocca, Piazza dell’Ateneo Nuovo, 1, 20126 Milan, MI Italy; 5 University of Messina, Piazza Pugliatti, 1, 98122 Messina, Italy; 6 Section of Endocrinology and Metabolism, Department of Medicine, Perugia University Medical School, Piazzale Gambuli, 1, 06129 Perugia, PG Italy; 7 University of Padua, Via 8 Febbraio 1848, 2, 35122 Padua, PD Italy; 8 University of the Eastern Piedmont, Via del Duomo, 6, 13100 Vercelli, VC Italy; 9 Sanofi, Milan, Italy; 10 OPIS s.r.l., Palazzo Aliprandi, Via Matteotti, 10, 20832 Desio, Italy

**Keywords:** Insulin glargine 300 U/mL, Titration, Self-titration, Hypoglycaemia

## Abstract

**Aims:**

The Italian Titration Approach Study (ITAS) demonstrated comparable HbA_1c_ reductions and similarly low hypoglycaemia risk at 6 months in poorly controlled, insulin-naïve adults with T2DM who initiated self- or physician-titrated insulin glargine 300 U/mL (Gla-300) in the absence of sulphonylurea/glinide. The association of patient characteristics with glycaemic and hypoglycaemic outcomes was assessed.

**Methods:**

This post hoc analysis investigated whether baseline patient characteristics and previous antihyperglycaemic drugs were associated with HbA_1c_ change and hypoglycaemia risk in patient- versus physician-managed Gla-300 titration.

**Results:**

HbA_1c_ change, incidence of hypoglycaemia (any type) and nocturnal rates were comparable between patient- and physician-managed arms in all subgroups. Hypoglycaemia rates across subgroups (0.03 to 3.52 events per patient-year) were generally as low as observed in the full ITAS population. Small increases in rates of 00:00–pre-breakfast and anytime hypoglycaemia were observed in the ≤ 10-year diabetes duration subgroup in the patient- versus physician-managed arm (heterogeneity of effect; *p* < 0.05).

**Conclusions:**

Comparably fair glycaemic control and similarly low hypoglycaemia risk were achieved in almost all patient subgroups with patient- versus physician-led Gla-300 titration. These results reinforce efficacy and safety of Gla-300 self-titration across a range of phenotypes of insulin-naïve people with T2DM.

**Clinical trial registration:**

EudraCT 2015-001167-39

**Supplementary Information:**

The online version of this article (10.1007/s00592-021-01675-0) contains supplementary material, which is available to authorized users.

## Introduction

In Italy, approximately 50% of people with type 2 diabetes (T2DM) have glycated haemoglobin (HbA_1c_) above the commonly recommended target of 7.0% (53 mmol/mol) [1]. Poor glycaemic control can be attributed to various causes including, but not limited to, delay in basal insulin (BI) initiation as well as its titration [2]. The latter may be due to a lack of time or resources available to the healthcare team, infrequent clinic visits, fear of injections, worries about hypoglycaemia, concerns about weight gain and clinical inertia [2]. Providing appropriate tools and support to allow people with diabetes to self-titrate their BI can improve glycaemic outcomes and benefit psychological well-being by allowing them to feel more in control of their condition [3, 4].

The Italian Titration Approach Study (ITAS) has shown that, with the assistance of a specialist diabetes nurse, people with T2DM who are insulin naïve and have poor glycaemic control can initiate and self-titrate their insulin glargine 300 U/mL (Gla-300) dose with comparable HbA_1c_ reductions and comparably low risk of hypoglycaemia versus physician-managed dose titration, in the absence of sulphonylureas (SU)/glinides [5]. The aim of the current post hoc subgroup analysis was to examine whether the results of ITAS are affected by baseline patient characteristics, such as age, renal function, diabetes duration, body mass index (BMI), HbA_1c_ and previous therapy.

## Materials and methods

In this post hoc analysis, the main study endpoints in ITAS (change in HbA_1c_, incidence and rates of hypoglycaemia at week 24) were assessed in the following subgroups of patients: age (< 65 and ≥ 65 years), class of estimated glomerular filtration rate (eGFR; < 60 and ≥ 60 ml/min/1.73 m^2^), known diabetes duration (≤ 10 and > 10 years), BMI (< 30 and ≥ 30 kg/m^2^), HbA_1c_ at baseline (≤ 8.5 and > 8.5% [≤ 69/ > 69 mmol/mol]) and number/type of previous antihyperglycaemic drugs (only metformin, metformin plus other antihyperglycaemic drug or no prior metformin) (Table 1).

**Table 1:** Clinical characteristics of patients in the ITAS subgroups by treatment arm (patient-managed and physician-managed BI titration, ITT population)

Parameter	Patient-managed *n* = 175	Physician-managed *n* = 180
*Age*		
< 65 years, *n* (%)	76 (43.4)	83 (46.1)
Mean (SD) age, years	55.1 (6.8)	54.9 (6.9)
≥ 65 years, *n* (%)	99 (56.6)	97 (53.9)
Mean (SD) age, years	71.1 (4.6)	71.3 (4.2)
*eGFR*		
< 60 mL/min/1.73 m^2^, n (%)	16 (9.1)	26 (14.4)
Mean (SD) eGFR, mL/min/1.73 m^2^	47.7 (10.5)	47.5 (9.8)
≥ 60 mL/min/1.73 m^2^, n (%)	157 (89.7)	150 (83.3)
Mean (SD) eGFR, mL/min/1.73 m^2^	90.3(13.5)	91.0(14.3)
*Disease duration*		
≤ 10 years, *n* (%)	80 (45.7)	75 (41.7)
Mean (SD) disease duration, years	5.9 (2.5)	5.0 (2.6)
> 10 years, *n* (%)	95 (54.3)	105 (58.3)
Mean (SD) disease duration, years	16.5 (6.7)	16.2 (7.0)
*HbA*_1c_		
≤ 8.5%, n (%)	72 (41.1)	67 (37.2)
Mean (SD) HbA_1c_, %	8.1 (0.3)	8.1 (0.3)
> 8.5%, n (%)	103 (58.9)	113 (62.8)
Mean (SD) HbA_1c_, %	9.2 (0.4)	9.2 (0.4)
*BMI*		
< 30 kg/m^2^, *n* (%)	101 (57.7)	99 (55.0)
Mean (SD) BMI, kg/m^2^	26.4 (2.3)	26.6 (2.3)
≥ 30 kg/m^2^, *n* (%)	74 (42.3)	81 (45.0)
Mean (SD) BMI, kg/m^2^	36.1 (5.4)	34.3 (4.1)
*Previous diabetes mediation*		
Metformin only, *n* (%)	47 (26.9)	51 (28.3)
Metformin + other, *n *(%)	113 (64.6)	116 (64.4)
No prior metformin, *n* (%)	15 (8.6)	13 (7.2)

*BMI* body mass index, *eGFR* estimated glomerular filtration rate, *SD* standard deviation

The study design and results of ITAS have been previously reported [5, 6]. Briefly, ITAS (EudraCT number: 2015-001167-39) was an Italian national, multicentre, phase IV, 24-week, open-label, randomised (1:1), parallel-group study (EudraCT number: 2015-001167-39), in insulin-naïve adults (≥ 18 years of age) with T2DM for ≥ 1 year and poor glycaemic control (HbA_1c_ ≥ 7.5 and ≤ 10% [≤ 53/ ≤ 86 mmol/mol]) on oral antihyperglycaemic drugs (OADs). SU/glinide treatment was discontinued at randomisation. The primary aim of ITAS was to assess non-inferiority in the change in HbA_1c_ over 24 weeks when Gla-300 dose titration was managed by the patient (with nurse assistance) or the physician, both using the same BI dose algorithm (Supplementary Table 1). Secondary outcomes included the incidence of ≥ 1 confirmed (≤ 70 mg/dL [≤ 3.9 mmol/L]) and/or severe hypoglycaemia, nocturnal (events in the time interval 00:00–05:59 h, as well as 00:00–pre-breakfast) [7, 8], or at any time of day (24 h), as well as the annualised rate of hypoglycaemic events. Severe hypoglycaemia was defined as an event that required the assistance of another person to actively administer carbohydrate or glucagon or to perform other resuscitative actions.

All participants provided written informed consent. The clinical trial protocol was approved by the appropriate local ethics committees and IRB/IEC. The study was conducted in accordance with the Declaration of Helsinki and the ICH guidelines for Good Clinical Practice (GCP) [9].

### Statistical analysis

Change in HbA_1c_ at week 24 was analysed in each subgroup using the same model described for the main study analysis, i.e. a linear mixed-effect model (LMEM) for repeated measures with titration approach and centre as fixed effects and the HbA_1c_ baseline value as covariate. For the primary study endpoint, homogeneity of the treatment effect among subgroups was assessed by including a subgroup by-treatment interaction in the LMEM.

Hypoglycaemia incidences were calculated as the proportion of patients with ≥ 1 hypoglycaemia over study period. Besides, the corresponding relative risk with the 95% confidence intervals (CI) by endpoint in each subgroup was presented. The Breslow–Day test was used to evaluate whether the risk of hypoglycaemic event was consistent (homogeneous) across the levels in each subgroup.

The hypoglycaemic events were analysed by means of a binomial (NB) regression analysis to obtain model-based estimates of annual rate (events per patient-year), rate ratios and* p* values for the treatment heterogeneity effect.

Differences in treatment effects across subgroups were only considered relevant if significant heterogeneity was observed (*p* < 0.05); however, due to the post hoc nature of this analysis, the inferential statistical tests will be used only for exploratory purposes.

## Results

### Baseline characteristics

Baseline characteristics of the overall ITAS population have been presented previously [5, 6]. The distribution of subgroups was balanced between treatment arms (Table 1), and sample size was also generally well balanced among subgroups within treatment arms with the exceptions of the eGFR < 60 mL/min/1.73 m^2^ and no prior metformin subgroups, which were noticeably smaller.

### Glycaemic control

**Fig. 1:**
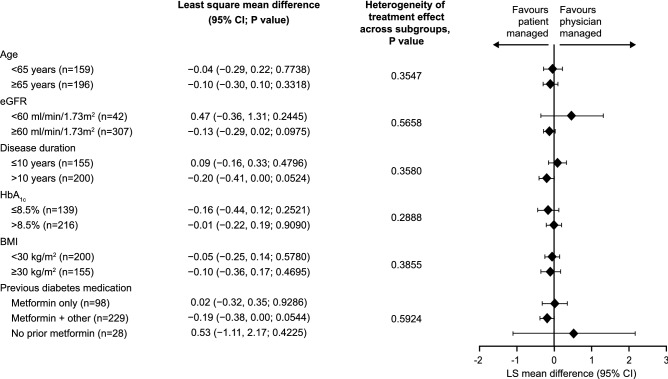
Differences in reduction of HbA_1c_ over 24 weeks between patient- and physician-managed BI titration in the subgroups of the ITAS population. Estimates and* p* values derived from a linear mixed-effect model (LMEM) for repeated measures

HbA_1c_ change at 6 months was comparable between the patient- and physician-managed arms, regardless of patient subgroup **(**Fig. 1, Supplementary Table 2). No evidence of heterogeneity of treatment effect was observed across any subgroup (*p* ≥ 0.05). The least squares (LS) mean differences (95% CI) between the patient- and physician-managed arms in each subgroup are reported in Fig. 1 and Supplementary Table 2.

### Hypoglycaemia

#### Incidence of confirmed (≤ 70 mg/dL [≤ 3.9 mmol/L]) and/or severe hypoglycaemic events

**Fig. 2:**
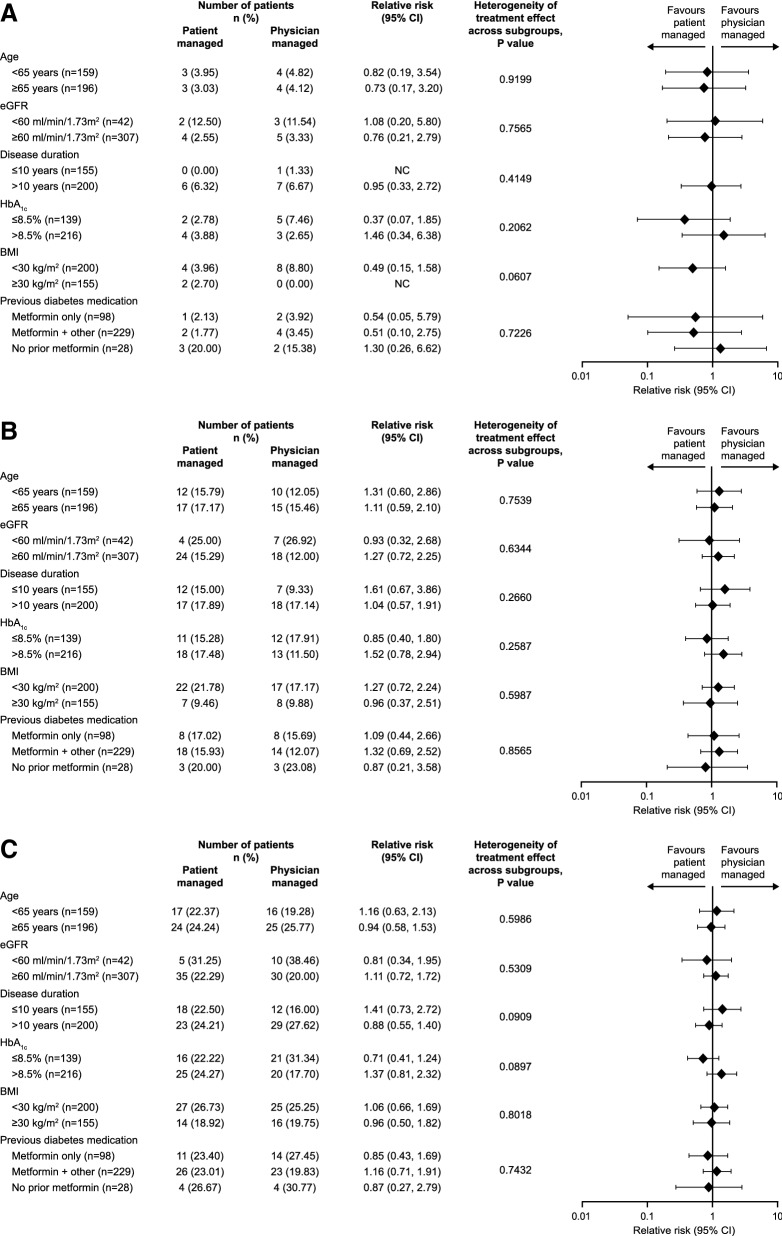
Incidence of confirmed (≤ 70 mg/dL, ≤ 3.9 mmol/L) and/or severe hypoglycaemia [(**a**) nocturnal (00:00–05:59 h), (**b**) 00:00 h–pre-breakfast, (**c**) any time] over 24 weeks in the subgroups of  the ITAS population, and differences between treatments (patient- and physician-managed BI titration). BMI, body mass index; CI, confidence intervals; eGFR, estimated glomerular filtration rate; NC, not calculable.* p* values derived from a Breslow–Day test of homogeneity

The risk of experiencing ≥ 1 nocturnal (00:00–05:59 h) or 00:00–pre-breakfast (expanded nocturnal window) confirmed (≤ 70 mg/dL [≤ 3.9 mmol/L]) and/or severe hypoglycaemic event was low in all subgroups, as already shown in ITAS [5]. Incidence of hypoglycaemia was unaffected by any of the assessed patient subgroups, with no evidence of heterogeneity of treatment effect observed (p ≥ 0.05). The relative risks (95% CI) of these nocturnal events in the patient-managed arm compared with the physician-managed arms are reported for each subgroup in Fig. 2a, b. Similarly, the incidence of any time confirmed (≤ 70 mg/dL [≤ 3.9 mmol/L]) and/or severe hypoglycaemia was not affected by any of the patient subgroups investigated (Fig. 2c). Severe hypoglycaemia in ITAS occurred in three out of 355 patients with a total of four events (two patients in the physician-managed arm) yielding a cumulative incidence of 0.8% [5].

#### Annualised rate of confirmed (≤ 70 mg/dL [≤ 3.9 mmol/L]) and/or severe hypoglycaemic events

The number of hypoglycaemic events and patient time at risk used to calculate the annualised rate of events are shown in Supplementary Table 3. Hypoglycaemia rates across subgroups were as low as observed in the full ITAS population [5].

**Fig. 3:**
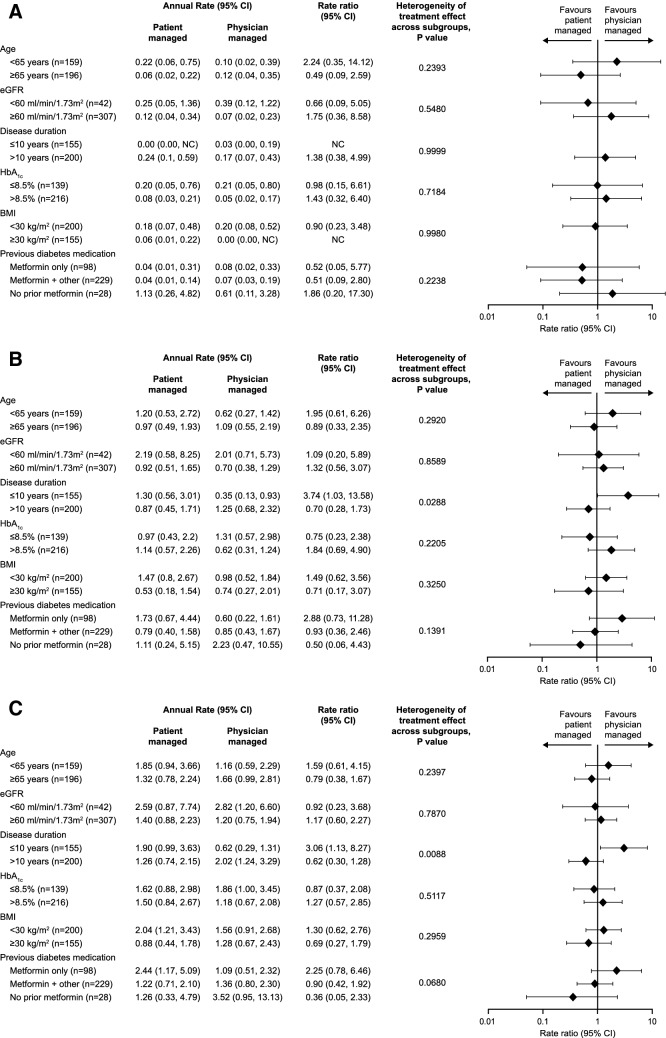
Annualised rates of confirmed (≤ 70 mg/dL, ≤ 3.9 mmol/L) and/or severe hypoglycaemia [(**a**) nocturnal (00:00–05:59 h), (**b**) 00:00 h–pre-breakfast, (**c**) any time] over 24 weeks in the subgroups of the ITAS population, and differences between treatments (patient- and physician-managed BI titration). BMI, body mass index; CI, confidence intervals; eGFR, estimated glomerular filtration rate; NC, not calculable. Annual rates presented as events per patient-year. Estimates and* p* values derived from a negative binomial (NB) regression model

No heterogeneity of treatment effect was observed for nocturnal (00:00–05.59 h) rates of hypoglycaemia in any subgroup (Fig. 3a). Statistically significant heterogeneity was observed for rates of the expanded window of nocturnal hypoglycaemia (00:00–pre-breakfast) in the subgroup of patients with disease duration ≤ 10 years due to higher rates of hypoglycaemic events in the patient-managed arm compared with the physician-managed arm (Fig. 3b). Similar results were observed for rates of any time hypoglycaemia in the same subgroup (Fig. 3c).

## Discussion

ITAS has previously shown that insulin-naïve people with T2DM, uncontrolled on OADs and/or non-insulin injectables can effectively and safely self-titrate Gla-300 (in the absence of SU/glinides treatment), with no differences in terms of HbA_1c_ reduction or risk of hypoglycaemia versus physician-managed titration [5]. The results of the present post hoc sub-analysis show that the improvement in glycaemic control was superimposable, and the incidence of hypoglycaemia was similarly low and not different between patient- and physician-managed titration of Gla-300 arms, regardless of age, renal function, diabetes duration, glycaemic control, BMI and metformin as the only treatment. The only statistically significant difference detected between the patient- and physician-managed arms was the annualised rate of hypoglycaemia (00:00 h–pre-breakfast, any time) in the subgroups with diabetes duration ≤ 10 years, which was higher in the patient-managed arm. However, the rates of non-severe hypoglycaemia were low and severe hypoglycaemia was virtually absent as observed in ITAS [5]. These results of similar incidence but greater rates of hypoglycaemia might be interpreted as a signal of more aggressive BI titration by some individual patients with shorter diabetes duration versus physicians. It is worth noting that a shorter diabetes duration has been reported as an indicator of greater likelihood to achieve HbA_1c_ < 7.0% in a large global population [10]. The small increase in rates of hypoglycaemia observed only in the diabetes duration subgroup of patient-managed titration in the present post hoc analysis of ITAS in the context of similar incidence should be weighted versus the advantage for the patients who effectively reduce HbA_1c_ with self-management of BI. One might imagine that implementation of BI self-management by patients might reduce the barriers of access to this treatment, which is simple, efficacious and safe, but time-consuming for physicians and expensive for the healthcare system.

Of note, in the present study BMI was not associated with different risks of hypoglycaemia between patient- and physician-managed titration. However, a lower BMI was associated with greater risk of hypoglycaemia in the total ITAS population, i.e. both patient- and physician-managed groups, as recently indicated in a study where lower BMI and lower C-peptide were associated with higher risk of hypoglycaemia upon initiation of BI in insulin-naïve people [11].

A number of trials have compared patient- versus physician-managed titration of Gla-300 with results largely consistent with the ITAS study [4, 12, 13]. Among these studies, the TAKE CONTROL trial, which investigated a mixed population of insulin-naïve and insulin-treated patients who continued SU/glinides, also included a post hoc subgroup analysis [4]. In this study, in subgroup < 65 and ≥ 65 years of age there was no heterogeneity of treatment effect for either HbA_1c_ reduction or incidence of any time hypoglycaemia, consistent with the results of the present ITAS subgroup analysis [14]. However, TAKE CONTROL did not report annualised rates of hypoglycaemia.

Limitations of the present study include the post hoc nature of the sub-analysis which remains an hypothesis-generating observation to be confirmed in ad hoc randomised controlled trials, and the numerical imbalance in the subgroups of eGFR (< vs. ≥ 60 ml/min/1.73 m^2^) and previous diabetes medication (metformin only, metformin + other and no prior metformin). Strength of this exploratory analysis derives from the clinical need to establish the applicability of the results of ITAS to subgroups of patients with quite different phenotypes, commonly observed in diabetes clinics. In this respect, the importance of subgroups analysis of glycaemic outcomes with BI initiation has recently been emphasised [15].

In conclusion, the present post hoc analysis of ITAS indicates that comparable glycaemic control and similarly low risk of hypoglycaemia were achieved with patient- and physician-managed Gla-300 titration, irrespective of assessed patient subgroups that were well balanced within treatment arms, except for the eGFR < 60 mL/min/1.73m^2^ and no prior metformin subgroups, which were noticeably smaller. A slightly higher rate of hypoglycaemia in the patient-managed arm than the physician-managed arm was detected in those with shorter disease duration, which deserves further investigation. Overall, the results suggest that in place of physician-managed titration of BI, patient-managed titration may be effective and generally safe across the several clinical varieties of T2DM phenotype. Thus, these findings agree with specific and effective reinforcement of diabetes self-management education and support [16] for earlier and more popular initiation and titration of BI.

## Supplementary Information

Below is the link to the electronic supplementary material.

Supplementary material 1 (DOCX 59 kb)

## Data Availability

Proposals relating to the data access should be directed to the corresponding author. To gain access, data requestors will need to sign a data access agreement.

## Abbreviations

BI: Basal insulin
BMI: Body mass index
Gla-300: Insulin glargine 300 U/mL
HbA_1c_: Glycated haemoglobin
GCP: Good Clinical Practice
ITAS: Italian Titration Approach Study
LMEM: Linear mixed-effect model
OADs: Oral antihyperglycaemic drugs
SU: Sulphonylureas
T2DM: Type 2 diabetes
