# Supplementary material for: Similar glycaemic control and risk of hypoglycaemia with patient- versus physician-managed titration of insulin glargine 300 U/mL across subgroups of patients with T2DM: a post hoc analysis of ITAS
Source: Acta Diabetol. 2021 Feb 14;58(6):789–96. doi: 10.1007/s00592-021-01675-0 (PMC8110495; doi:10.1007/s00592-021-01675-0)
Supplement: Supplementary file 1 — Supplementary material 1 (DOCX 59 kb) [file 592_2021_1675_MOESM1_ESM.docx]

**Supplementary Materials**

**Similar glycaemic control and risk of hypoglycaemia with patient- vs physician-managed titration of insulin glargine 300 U/mL across subgroups of patients with T2DM: a post-hoc analysis of ITAS**

A. Giaccari^a^, R.C. Bonadonna^b^, R. Buzzetti^c^, G. Perseghin^d^, D. Cucinotta^e^, C. Fanelli^f^, A. Avogaro^g^, G. Aimaretti^h^, M. Larosa^i^, V. Pagano^j^, G.B. Bolli^f^

*^a^Fondazione Policlinico Universitario A. Gemelli IRCCS, Rome and Università Cattolica del Sacro Cuore, Rome, Italy; ^b^Division of Endocrinology and Metabolic Diseases and Department of Medicine and Surgery, University of Parma and AOU of Parma, Italy; ^c^Sapienza University of Rome, Piazzale Aldo Moro, 5, 00185 Roma RM, Italy; ^d^University of Milan Bicocca, Piazza dell'Ateneo Nuovo, 1, 20126 Milano MI, Italy; ^e^University of Messina, Piazza Pugliatti, 1 - 98122 Messina, Italy; ^f^Section of Endocrinology and Metabolism, Department of Medicine, Perugia University Medical School, Piazzale Gambuli, 1, 06129 Perugia PG, Italy; ^g^University of Padua, Via 8 Febbraio 1848, 2, 35122 Padova PD, Italy; ^h^University of the Eastern Piedmont,* *Via del Duomo, 6, 13100 Vercelli VC, Italy; ^i^Sanofi, Milan, Italy; ^j^OPIS s.r.l., Palazzo Aliprandi, Via Matteotti, 10 – 20832 Desio, Italy*

**Corresponding author:** Andrea Giaccari, Andrea.Giaccari@unicatt.it, Fondazione Policlinico Universitario A. Gemelli IRCCS, Rome and Università Cattolica del Sacro Cuore, Rome, Italy

**Supplementary Table 1.** Titration algorithm of basal insulin Gla-300 used by patients and physicians

|  | **Gla-300 dose adjustment (Units)^†^** | |
| --- | --- | --- |
| Fasting SMBG* | **Patient-managed** | **Physician-managed** |
| **>180 mg/dL (>10 mmol/L)** | +4 | |
| **110–180 mg/dL (6.1–10 mmol/L)** | +2 | |
| **80–110 mg/dL (4.4–6.1 mmol/L)** | No change | |
| **<80 mg/dL (<4.4 mmol/L)** | -2 | |
| **<54 mg/dL or occurrence of ≥2 symptomatic or 1 severe episode of hypoglycaemia in the preceding week** | Contact physician | At physician’s discretion |

^†^Dose should be adjusted every 3–4 days to achieve a target range for fasting SMBG of 80–100 mg/dL.

*Participants self-administered Gla-300 as a daily subcutaneous injection at the same time between dinner and bedtime each evening. Gla-300 was administered at a starting dose of 0.2 U/kg, as per label, and then adjusted if needed – based on the median SMBG values measured on the previous three consecutive days – not more often than every 3–4 days to achieve a fasting SMBG of 80–110 mg/dL, using the same algorithm in both study groups. Fasting SMBG values were measured on 3 consecutive days, of which the last was the day when titration is to occur. Visits/contacts were weekly until Week 12 and bi-weekly until week 24. Patients self-managing insulin titration received a special education session regarding the titration algorithm from the study nurse, who monitored but did not influence titration according to the algorithm. In the physician-managed titration arm, dose adjustments were made during visits/contacts with the physician.

**Supplementary Table 2.** Reductions in HbA_1c_ over 24 weeks by subgroup

| **Baseline parameter** | **Mean (SD) baseline HbA_1c_, %** | | **Mean (SD) Week 24  HbA_1c_, %** | | **Least square estimate* HbA_1c_ reduction**  **Week 24 vs baseline (95% CI)** | |
| --- | --- | --- | --- | --- | --- | --- |
|  | Patient  managed | Physician managed | Patient  managed | Physician managed | Patient  managed | Physician managed |
| Age |  |  |  |  |  |  |
| <65 years (n=159) | 8.77 (0.69) | 8.94 (0.60) | 7.04 (0.65) | 7.17 (0.80) | −1.83 (−2.02, −1.64) | −1.80 (−1.99, −1.60) |
| ≥65 years (n=196) | 8.76 (0.65) | 8.71 (0.65) | 7.21 (0.74) | 7.36 (0.78) | −1.44 (−1.60, −1.28) | −1.34 (−1.49, −1.18) |
| eGFR |  |  |  |  |  |  |
| <60ml/min/1.73m^2^ (n=42) | 8.85 (0.63) | 8.72 (0.57) | 7.41 (1.02) | 7.22 (0.82) | −1.17 (−1.80, −0.54) | −1.64 (−2.07, −1.22) |
| ≥60ml/min/1.73m^2^ (n=307) | 8.76 (0.67) | 8.83 (0.65) | 7.12 (0.66) | 7.28 (0.79) | −1.66 (−1.79, −1.54) | −1.53 (−1.65, −1.40) |
| Disease duration |  |  |  |  |  |  |
| ≤10 years (n=155) | 8.86 (0.64) | 8.88 (0.61) | 7.12 (0.74) | 7.12 (0.78) | −1.60 (−1.78, −1.41) | −1.69 (−1.88, −1.50) |
| >10 years (n=200) | 8.69 (0.69) | 8.77 (0.65) | 7.16 (0.68) | 7.38 (0.79) | −1.57 (−1.74, −1.40) | −1.37 (−1.52, −1.21) |
| HbA_1c_ |  |  |  |  |  |  |
| ≤8.5 % (n=139) | 8.10 (0.29) | 8.13 (0.29) | 6.93 (0.56) | 7.21 (0.79) | −1.17 (−1.38, −0.97) | −1.01 (−1.20, −0.82) |
| >8.5 % (n=216) | 9.24 (0.40) | 9.23 (0.38) | 7.28 (0.76) | 7.31 (0.80) | −1.89 (−2.05, −1.73) | −1.88 (−2.04, −1.72) |
| BMI |  |  |  |  |  |  |
| <30 kg/m^2^ (n=200) | 8.73 (0.68) | 8.82 (0.67) | 7.11 (0.65) | 7.23 (0.74) | −1.64 (−1.79, −1.50) | −1.59 (−1.73, −1.44) |
| ≥30 kg/m^2^ (n=155) | 8.82 (0.65) | 8.81 (0.60) | 7.18 (0.78) | 7.33 (0.86) | −1.62 (−1.82, −1.41) | −1.52 (−1.72, −1.32) |
| Previous antihyperglycaemic drugs |  |  |  |  |  |  |
| Metformin only (n=98) | 8.77 (0.62) | 8.76 (0.62) | 7.05 (0.75) | 7.01 (0.62) | −1.74 (−1.98, −1.49) | −1.75 (−2.00, −1.50) |
| Metformin + other (n=229) | 8.77 (0.67) | 8.86 (0.64) | 7.16 (0.72) | 7.40 (0.83) | −1.60 (−1.76, −1.44) | −1.41 (−1.56, −1.27) |
| No prior metformin (n=28) | 8.72 (0.78) | 8.68 (0.63) | 7.29 (0.43) | 7.12 (0.85) | −1.23 (−2.24, −0.21) | −1.75 (−2.60, −0.91) |

BMI, body mass index; CI, confidence interval; eGFR, estimated glomerular filtration rate; HbA_1c_, glycated haemoglobin; SD, standard deviation. *Estimates and p values derived from a Linear Mixed-Effect Model (LMEM) for repeated measures.

**Supplementary Table 3.** Total number of confirmed (≤70 mg/dL, ≤3.9 mmol/L) and/or severe (A) nocturnal (00:00–05:59 h), (B) 00:00 h–pre-breakfast and (C) any time hypoglycaemia events, and total patient-years effectively spent in the 24 week study for each subgroup with the two treatments (patient- and physician-managed Gla-300 titration)

**A**

| **Baseline parameter** | **Patient managed** | | **Physician managed** | |
| --- | --- | --- | --- | --- |
|  | Events, n | Patient-years in study | Events, n | Patient-years in study |
| Age |  |  |  |  |
| <65 years | 8 | 36.83 | 4 | 40.69 |
| ≥65 years | 3 | 49.17 | 6 | 47.17 |
| eGFR |  |  |  |  |
| <60 ml/min/1.73m^2^ | 2 | 7.86 | 5 | 12.69 |
| ≥60 ml/min/1.73m^2^ | 9 | 77.11 | 5 | 73.52 |
| Disease duration |  |  |  |  |
| ≤10 years | 0 | 39.66 | 1 | 36.70 |
| >10 years | 11 | 46.34 | 9 | 51.16 |
| HbA_1c_ |  |  |  |  |
| ≤8.5 % | 7 | 34.75 | 7 | 32.78 |
| >8.5 % | 4 | 51.24 | 3 | 55.09 |
| BMI |  |  |  |  |
| <30 kg/m^2^ | 9 | 50.11 | 10 | 48.31 |
| ≥30 kg/m^2^ | 2 | 35.89 | 0 | 39.56 |
| Previous antihyperglycaemic drugs |  |  |  |  |
| Metformin only | 1 | 23.20 | 2 | 24.27 |
| Metformin + other | 2 | 55.84 | 4 | 57.37 |
| No prior metformin | 8 | 6.96 | 4 | 6.23 |

**B**

| **Baseline parameter** | **Patient managed** | | **Physician managed** | |
| --- | --- | --- | --- | --- |
|  | Events, n | Patient-years at risk | Events, n | Patient-years at risk |
| Age |  |  |  |  |
| <65 years | 45 | 36.83 | 25 | 40.69 |
| ≥65 years | 47 | 49.17 | 53 | 47.17 |
| eGFR |  |  |  |  |
| <60 ml/min/1.73m^2^ | 17 | 7.86 | 26 | 12.69 |
| ≥60 ml/min/1.73m^2^ | 71 | 77.11 | 52 | 73.52 |
| Disease duration |  |  |  |  |
| ≤10 years | 52 | 39.66 | 13 | 36.70 |
| >10 years | 40 | 46.34 | 65 | 51.16 |
| HbA_1c_ |  |  |  |  |
| ≤8.5 % | 34 | 34.75 | 44 | 32.78 |
| >8.5 % | 58 | 51.24 | 34 | 55.09 |
| BMI |  |  |  |  |
| <30 kg/m^2^ | 73 | 50.11 | 48 | 48.31 |
| ≥30 kg/m^2^ | 19 | 35.89 | 30 | 39.56 |
| Previous antihyperglycaemic drugs |  |  |  |  |
| Metformin only | 40 | 23.20 | 16 | 24.27 |
| Metformin + other | 44 | 55.84 | 48 | 57.37 |
| No prior metformin | 8 | 6.96 | 14 | 6.23 |

**C**

| **Baseline parameter** | **Patient managed** | | **Physician managed** | |
| --- | --- | --- | --- | --- |
|  | Events, n | Patient-years at risk | Events, n | Patient-years at risk |
| Age |  |  |  |  |
| <65 years | 69 | 36.83 | 47 | 40.69 |
| ≥65 years | 64 | 49.17 | 80 | 47.17 |
| eGFR |  |  |  |  |
| <60 ml/min/1.73m^2^ | 20 | 7.86 | 36 | 12.69 |
| ≥60 ml/min/1.73m^2^ | 108 | 77.11 | 89 | 73.52 |
| Disease duration |  |  |  |  |
| ≤10 years | 75 | 39.66 | 23 | 36.70 |
| >10 years | 58 | 46.34 | 104 | 51.16 |
| HbA_1c_ |  |  |  |  |
| ≤8.5 % | 57 | 34.75 | 62 | 32.78 |
| >8.5 % | 76 | 51.24 | 65 | 55.09 |
| BMI |  |  |  |  |
| <30 kg/m^2^ | 101 | 50.11 | 76 | 48.31 |
| ≥30 kg/m^2^ | 32 | 35.89 | 51 | 39.56 |
| Previous antihyperglycaemic drugs |  |  |  |  |
| Metformin only | 56 | 23.20 | 28 | 24.27 |
| Metformin + other | 68 | 55.84 | 77 | 57.37 |
| No prior metformin | 9 | 6.96 | 22 | 6.23 |

BMI, body mass index; CI, confidence interval; eGFR, estimated glomerular filtration rate; HbA_1c_, glycated haemoglobin; NC, not calculable.

Patient time at risk was computed in years elapsed between screened date and the visit at week 24.
